# Evaluation of the Dietary Habits of Polish and Greek University Students in the Context of the Health Benefits of Their Diets

**DOI:** 10.3390/nu16223819

**Published:** 2024-11-07

**Authors:** Patrycja Widłak, Marzena Malara, Łukasz Tomczyk, Aspasia Dania, Georgia Panagiotakou, Georgia Papoulia

**Affiliations:** 1 Department of Human Biology, The Faculty of Physical Education, Józef Piłsudski University of Physical Education in Warsaw, 00-968 Warsaw, Poland; marzena.malara@awf.edu.pl; 2 Department of Food Quality and Safety Management, Faculty of Food Science and Nutrition, Poznan University of Life Sciences, 60-637 Poznan, Poland; lukasz.tomczyk@up.poznan.pl; 3 School of Physical Education and Sport Science, National and Kapodistrian University of Athens, 17237 Athens, Greece; adania@phed.uoa.gr (A.D.); panagiotakoutzina1@gmail.com (G.P.); gogopapoul@gmail.com (G.P.)

**Keywords:** nutrition, healthy diet, lifestyle

## Abstract

**Background/Objectives**: Eating habits and behaviors play a central role in maintaining health and overall well-being. In the case of young students, they affect not only appearance and general mood but also cognitive ability, concentration, and broader learning ability. For this reason, it is essential for young people to have well-balanced and wholesome diets. **Methods**: The study included 186 Polish and 136 Greek university students majoring in sports. The research tool was the validated survey questionnaire FFQ-6, and the data collected were additionally used to calculate the healthy diet index (pHDI-10). **Results**: Based on the analysis of the collected data, the dietary habits of the students surveyed were assessed, taking into account, among other things, the frequency of consumption of sweets, salty snacks, processed foods, and sweetened drinks, but also healthy products, including vegetables and fruit or vegetable and fruit juices. The data collected made it possible to determine an index of the health value of the participants’ diet. **Conclusions**: The analysis of the collected data led to the conclusion that, more often than their Greek peers, Polish university students tend to reach for sweets, salty snacks, or sweetened drinks. Greeks were more likely to choose fruit and vegetables and vegetable and fruit juices. However, among both Poles and Greeks, the index of the health-promoting value of the diet is low, indicating a high risk of diet-related diseases of lifestyle. Similar trends can be observed among students of almost all nationalities, which makes it reasonable to consider the implementation of appropriate educational programs on nutrition and healthy lifestyle principles in general.

## 1. Introduction

Eating behaviors result from eating patterns and daily habits developed over time by individuals. Examining the available evidence on diet helps to understand the relationship between dietary habits and health outcomes. Good eating habits are one of the most important elements of lifestyle. They are particularly important for young people during the intensive education and development periods. An adequate supply of nutrients, vitamins, and minerals during this period has an impact not only on physical development, the building of muscle mass, or the peak of bone mass but also on intellectual abilities, including the ability to learn [1,2,3,4,5,6,7,8,9,10,11,12,13,14].

For many young people, tertiary education is when they leave the family home and begin living independently. Lack of parental control, frequent socializing with friends, a limited budget, and often a lack of time or facilities to prepare meals mean that young people’s eating habits frequently change considerably. The quality and number of meals change, main meals are replaced by sweet or salty snacks, and fast food consumption often increases. As shown in the study by Moscatelli et al., negative changes do not necessarily have to be the rule. In the multicenter study they cited, it was observed that students in Turkey, Romania, Croatia, and Lebanon exhibited a greater tendency toward unhealthy behaviors. On the other hand, students from Spain and Italy tended to engage in health-promoting behaviors [15].

On the other hand, energy drinks high in sugar, caffeine, and taurine are consumed by young people in an attempt to compensate for sleep deprivation. College is also a time for establishing new relationships, accompanied by numerous social meetings that also encourage changes in eating habits, not only in the daily rhythm of food intake but also in the type of food. Social gatherings are often accompanied by high intakes of salty and sweet snacks and various alcoholic drinks [16,17,18,19,20,21,22,23,24,25].

An additional factor that may influence dietary changes during the university period seems to be the economic aspect. The need to manage a usually limited budget and a lack of time, while simultaneously meeting multiple needs, may lead to the decision to purchase lower-quality and more readily available products.

A separate aspect is meal planning and shopping for meal preparation. It has been observed that students who remain in the family home during their studies show a significantly lower tendency to change their eating habits in a negative direction, as they remain under family care, and the responsibility for meal planning and shopping often does not fall on them. On the other hand, students who leave the family home tend to experience a greater decline in their dietary habits [15].

Irregular and incomplete meals can affect several aspects of physical and mental health, and frequent consumption of convenience foods, highly processed foods, and alcohol negatively affects general health, well-being, life satisfaction, sleep quality, concentration, memory, and learning ability, which are particularly taxed during the study process [26,27,28,29,30].

Eating habits and dietary choices vary from country to country. However, the principles of healthy eating have a fairly universal and international dimension. The cultural specificity of eating patterns, ingredient selection and availability, seasonality of foods, or social habits, especially among the younger members of society worldwide, are increasingly being displaced by the prevalence, availability, and affordability of fast food, sugary snacks, and energy drinks. This is leading to an increase in the prevalence of obesity, type II diabetes, cardiovascular disease, and a range of other lifestyle diseases [18,20,22,25,27,29,31,32,33,34].

The school year seems to be a very good time to educate young people about health. After previous stages of education, young people already have sufficient theoretical knowledge of the principles of a healthy lifestyle, are aware of the consequences of bad health habits, and, at the same time, are mature enough to understand the long-term consequences of their health behavior choices. Education to achieve this goal therefore seems to be highly justified [19,20,21,22,24,32,33,34].

The study aimed to compare and evaluate the dietary habits of Polish students at the Józef Piłsudski University of Physical Education in Warsaw (Poland) and Greek students at the Kapodistrian University of Athens (Greece) in terms of health behavior, body weight, and the health-promoting value of their diets. A survey on the availability of health-promoting and unhealthy products revealed that participants from both environments, despite significant differences in the characteristics of local cuisine, have similar access to such products. Therefore, their dietary choices are primarily influenced by their personal decisions rather than by limitations in the availability of health-promoting or unhealthy products. For this reason, it was assumed that the study would be exploratory, and no research hypotheses were formulated.

## 2. Materials and Methods

The study material consisted of 186 Polish students studying at the Józef Piłsudski University of Physical Education in Warsaw and 137 Greek students studying at the Kapadostrias University in Athens. Students at both universities studied sports.

The study used the Food Frequency Questionnaire with 6 answers (FFQ-6), consisting of 62 questions on the consumption of different product groups in the last 12 months, divided into 8 sections: sweets and snacks, dairy products and eggs, cereals, fats, fruit, vegetables and cereals, meat products and fish, and beverages [35]. Based on the obtained data, it is possible to identify individuals with varying levels of consumption frequency for specific products and/or to distinguish characteristic food consumption patterns within the evaluated population.

The original questionnaire was written in Polish. For the study, it was also translated into Greek, using a nomenclature referring to analogous product groups and the products available in both countries. The data collected allowed an analysis of the dietary habits of the respondents in general. Taking into account the varying frequency of consumption, including health-promoting products, the decision was taken to also analyze the collected data in terms of the pro-health value of the diet using the Pro-Healthy Diet Index (pHDI-10) (Table 1). The pHDI-10 Index is used to calculate the health-promoting properties of a diet and is based on the consumption of 10 food groups, whose frequency of intake was assessed using the FFQ-6 questionnaire. These food groups were derived from the FFQ-6 based on current guidelines on healthy eating principles. The collected data were applied to the following formula: Pro-Healthy Diet Index (pHDI-10, in points) = (100/20) × the sum of the consumption frequency of 10 food groups (times/day) [36,37].

Due to the nature of the research tool, which was a questionnaire, it was not possible to assess factors such as the body composition of the participants. Therefore, the study was limited to the BMI index, which was used to evaluate the participants’ body weight. Body mass index was calculated as kg/m^2^ and categorized into underweight (BMI < 18.5), normal weight (18.5–24.9), overweight (25–29.9), and obese (≥30), following the international BMI classification. BMI was calculated by dividing the body mass in kilograms by the square of height in meters. The international classification was applied, where a BMI value of <18.5 indicates underweight, 18.5–24.9 is normal, 25–29.9 is overweight, and ≥30 is obesity [22]. The obtained values were compared with the pHDI-10 to assess which students (underweight, normal weight, overweight, or obese) had the most health-promoting diets.

All students were provided with an online version of the questionnaire in Polish or Greek using MS Forms. Before distributing the questionnaire to respondents, a cross-translation of the text was conducted in both groups to increase the reliability of the questionnaire. Both research teams discussed the consistency of the meaning of the questions in the Polish and Greek versions. The recruitment of participants was carried out randomly. Participation in the study was voluntary, and before completing the questionnaire, participants were informed about the study’s purpose, the intended use of the collected data, and the anonymity of the study. The study was conducted based on the approval of the Ethics Committee of the Senate of the Józef Piłsudski Academy of Physical Education in Warsaw, which the Kapadistrias University of Athens accepted. Inclusion criteria were confirmation of being a student at the university, participating in the study, and informed consent for participation, confirmed by voluntary completion of the study questionnaire. Exclusion criteria were not being a student of the participating universities and refusing to participate in the study.

Statistical analysis and visualization of the collected data were performed using Statistica 13.3. To characterize the study groups, the mean, median, and standard deviation were calculated for the representatives of both nationalities. Pearson’s chi-squared test was used to test the relationship between qualitative variables such as frequency of consumption of selected food meals (Never or rarely, Once a month or less, Several times a month, Several times a week, and Daily Several times a day) and country of origin (Poland, Greece). The significance level was set at α = 0.05. The following abbreviations and symbols were used: *p*—statistical significance; if *p* < 0.05, there was a significant difference between the groups.

Additionally, Correspondence Analysis (CA) was performed. This method is used to examine relationships between categorical variables, visualized on a two-dimensional contingency plot. In this study, it was carried out by analyzing and visualizing food groups that have similar or different “profiles”, meaning the relative frequency of consumption depending on the origin of the study group (Poland—1, Greece—2). The CA plot presents a visualization of the chi-square deviations (inertia) of consumption frequencies from their respective average profile.

## 3. Results

Table 2 shows the characteristics of the study participants. It was found that the vast majority of respondents from both nationalities—approximately 80%—had a normal BMI value, which is significant information in the context of the analyzed parameters.

Based on the data collected in Table 3, there were statistically significant differences between Polish and Greek students in the intake of biscuits and cakes, salty snacks, dairy products, whole meal and refined bread, oil and butter, vegetables and fruit, and poultry and red meat: Polish students were much more likely to consume refined and processed products, while Greek students were more likely to consume dairy products, oil, and vegetables.

The frequency of consumption of selected types of beverages among the participants is summarized in Table 4. Statistically significant differences were observed in the consumption of vegetable and vegetable–fruit juices and sweetened beverages, as well as beer, wine, and alcoholic drinks by Polish and Greek respondents. Polish students consumed sweetened drinks, beer, wine, and soft drinks more frequently than their Greek peers (several times a month), while Greek students consumed vegetable and vegetable–fruit juices daily and wine and soft drinks several times a week. However, there were no statistically significant differences in the consumption of vodka and spirits.

Based on the data collected in Table 5, it can be concluded that a low probability of a favorable level of the pHDI-10 index is prevalent among the respondents. Only slightly more than 4% of the respondents were in the medium probability range of a healthy diet, and none of the study participants were in the high probability range. It can therefore be assumed that the distribution of the pHDI-10 healthy diet index in the study group was unfavorable.

Analysis of the data presented in Table 6 shows that there was no statistically significant difference between Polish and Greek students in the low probability of a healthy diet, while in the moderate category, the distribution of the data was the same, but this group was very small among both Poles and Greeks. None of the respondents of either nationality was in the high likelihood category.

Table 7 shows the percentage of Polish and Greek students surveyed by pHDI-10 index ranges with regards to their nutritional status as determined by BMI. For both Polish and Greek students, no statistically significant correlations were found between pHDI-10 index ranges and BMI categories. The proportions of respondents characterized as having a low and moderate likelihood of their diet being healthy are similar for each BMI category in both countries. However, there is a predominance of respondents in the low category, indicating a low index of their diet being healthy.

**Figure 1:**
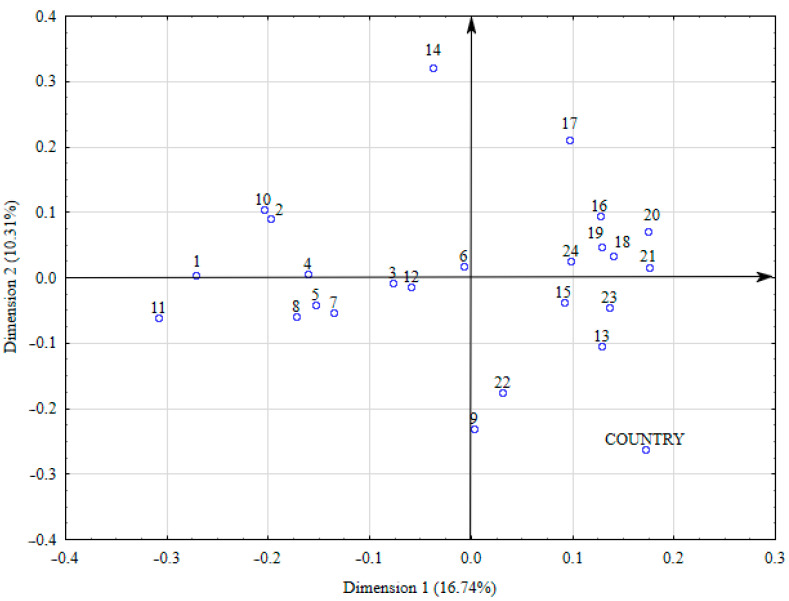
Correspondence table of the relationship between the consumption frequency of selected food products and the origin of the respondents. Explanation of the numbers in the figure: 1—Sugar for sweetening beverages; 2—Non-chocolate candies; 3—Biscuits and cookies; 4—Salty snacks; 5—Refined bread; 6—Refined fine-grain groats; 7—Other animal fats; 8—Sausages, various types; 9—Red meat, e.g., pork, beef, veal; 10—Energy drinks; 11—Sweetened beverages; 12—Vodka and high-proof alcohol; 13—Milk and natural milk beverages; 14—Natural cottage cheese; 15—Eggs and egg-based dishes where eggs are the primary ingredient; 16—Whole grain or seeded bread; 17—Coarse, unrefined groats; 18—Fruits, of all kinds; 19—Vegetables, of all kinds; 20—Dried legumes and seeds; 21—Nuts and nut butters; 22—Poultry and rabbit meat; 23—Lean fish; 24—Fatty fish.

To illustrate multidimensional deviations (inertia) reduced to the two most informative dimensions, a correspondence table/plot was created (Figure 1). The distance between the row and column profile points, as well as the direction in which they are displaced from the origin, indicates their relationship (the relationship is stronger if the points are located in similar directions from the origin). The first axis (horizontal) accounts for the largest share of inertia (16.74%) and is therefore the best dimension for explaining most of the variation between the rows. On the plot, this axis distinguishes two groups: on the far left, mostly products considered unhealthy, and on the right, products considered healthy.

## 4. Discussion

Good nutrition is a key factor in the maintenance of good health and the prevention of lifestyle diseases. It is extremely important at all stages of life, but it is especially relevant for young people, who are undergoing physical development while at the same time undergoing considerable mental stress as a result of the educational process. The considerable energy expenditure caused by both physical and mental exertion must be balanced by an appropriate diet rich in essential nutrients. A properly composed diet not only meets the needs for essential building components and energy but also helps maintain an optimal body weight. Among the study participants, the vast majority of students of both nationalities had a normal body weight, with mean values in the lower half of the range predicted for a normal body weight. Similar observations were made by Król et al. [38], who studied Polish, Portuguese, and Belarusian university students, and by Alghadir et al. [39] in a study of Saudi, Egyptian, Indian, Pakistani, and Afghan students. García-Meseguer’s [40] team, who carried out a study involving Spanish students, Dakanalis et al. [41], who analyzed the dietary habits of Greek students from different universities, and Spanos and Hankey [31], who compared Scottish and Greek students, also recognized this trend. However, in all these cases, the researchers found that the average BMI was at or above 22. This means that respondents from all groups were in the top half of the norm on the scale. It can therefore be seen that the average BMI of students of different nationalities is within the normal range, but the BMI of sports university students is lower.

A student lifestyle, due to its heavy course load, favors the consumption of readily available processed and energy-dense foods. Based on the data collected, the consumption of processed foods, sweets, salty snacks, and sweetened beverages was found to be more common among Polish than Greek students. Vegetables, dairy products, and fruit and vegetable juices were preferred by Greek students. Observations indicating the unfavorable eating habits of students have been made by, among others, Park and Papadaki [17], who conducted a study among British students who used food vending machines choosing sweets and salty snacks, or Spanos and Hankey [31], who examined Scottish and Greek students. It is noteworthy that the second group of researchers made similar observations about the dietary choices of Greeks to those presented in the paper, with Greek students less likely to choose salty snacks and sweets than their Scottish peers. An analysis of other studies shows that Malaysian students have a preference for sweet snacks and fruit [19]. Chinese students, on the other hand, have good eating habits [22]. They eat large amounts of green vegetables, fish, and meat, and very few fast food products compared to international students studying in China. This may be indicative of habits acquired before university. Yahia et al. [20] showed in their study that despite the growing trend of fast food in Lebanon, students still have quite good eating habits. More than 30% of the respondents reported consuming colorful vegetables and fruits daily. Similar observations were made by El Ansari et al. [24] who surveyed Finnish students. They found a rather high preference for sweets but at the same time a high consumption of vegetables. However, not in all areas of the world do students eat healthily. Rivera Medina et al. [23], in their study, found that students in Puerto Rico preferred fried foods and fizzy drinks and did not eat enough fruit, vegetables, and dairy products. Martinez-Perez et al. [18], in a study of Spanish students, also found a preference for sweets, salty snacks, and sugary drinks, which were preferred more often than foods described as healthy. However, this study also highlighted an important factor that may determine unhealthy food choices: when analyzing students’ alcohol consumption, a common trend, similar to that observed in this paper, is that students tend to consume low levels of or no alcohol. Spirits are not very popular among students.

The analysis of the healthy diet index showed that the dietary habits of the respondents urgently need improvement. More than 90% of the students were in the group with a low healthy diet index. This means a high predisposition to diet-related lifestyle diseases. A similar trend was shown in Polish students as well as those from other academic centers: Spanish, Chilean, or Romanian [42,43,44,45,46,47,48,49]. All the respondents came from sports universities and their average BMI was within the normal range. This indicates that students, including those from sports universities, where the level of nutritional education seems to be very high, need to be included in a preventive program aimed at preventing diet-related lifestyle diseases.

## 5. Conclusions

The analysis of the collected data leads to the conclusion that the respondents of both Polish and Greek nationality are predominantly of normal body weight. A small percentage of the participants of both groups had a BMI indicating overweight/obesity, but given that they were students of universities of physical education, such a result could also be due to high muscle mass. To enhance the credibility of the study, an analysis of participants’ body composition and a more comprehensive assessment of their health status would be necessary. However, the study was a pilot, and the research tool used—a questionnaire—did not allow for the collection of such data. It was also noted that Polish students are more likely to choose sweets, salty snacks, and processed foods compared to their Greek peers who are more likely to choose vegetables and dairy products. They are more likely to opt for sweetened drinks, while Greeks are more likely to choose vegetables, fruit, and vegetable juices. Compared to the Poles, Greeks were slightly more likely to consume low-proof spirits, while no significant difference was found between the study groups for the consumption of high-proof spirits. Eating habits indicating a low probability of a healthy diet index are prevalent among the respondents. Few participants, in similar proportions for both nationalities, showed a moderate probability of a healthy diet, and no participants showed a low probability of a healthy diet. Therefore, it can be concluded that the diet of the vast majority of participants of both nationalities has characteristics predisposing to the development of lifestyle diseases and therefore in need of intervention. None of the study groups showed any correlation between BMI and the probability of a healthy diet index.

## Figures and Tables

**Table 1:** Pro-Healthy Diet Index interpretation.

Probability of Pro-Healthy Diet Properties	Range (Times Per Day)	Range (Points)
low	0.00–6.66	1–33
moderate	6.67–13.33	34–66
high	13.34–20	67–100

**Table 2:** Characterization of study participants.

Country	Age (Years)	Height (Centimeters)	Weight (Kilograms)
**Poland** **N (186)**	19.7 ± 0.47	182 ± 8.77	66.3 ± 13.04
	
**Greece** **N (137)**	22.3 ± 5.27	170.7 ± 8.83	64.1 ± 11.35
	
	BMI	N	N
**Poland**	Normal BMI [%]	148	79.56
	Overweight/Obese [%]	32	17.20
	Underweight [%]	6	3.22
**Greece**	Normal BMI [%]	114	83.21
	Overweight/Obese [%]	16	11.67
	Underweight [%]	7	5.10

**Table 3:** Characteristics of the frequency of consumption of selected food groups among participants.

Food Products		Country		Total N	*p* *	chi^2^
		Poland	Greece			
Biscuits and cakes	Several times a day	100.00%	0.00%	1	0.0006 **	22.41
(e.g., shortbread, semi-shortbread, cream-filled, fruit, yeast-based, cheesecakes, donuts, poppy seed cakes, pastries, muffins, etc.)	Daily	20.00%	80.00%	5		
	Several times a week	65.85%	34.15%	41		
	Several times a month	67.63%	32.64%	144		
	Once a month or less	48.67%	51.33%	58		
	Never or almost never	26.32%	73.68%	19		
Salty snacks (e.g., chips, salted crisps, crackers, pretzels, etc.)	Several times a day	0.00%	0.00%	0	0.0001 **	64.71
	Daily	100.00%	0.00%	1		
	Several times a week	72.73%	27.27%	44		
	Several times a month	65.00%	35.00%	49		
	Once a month or less	52.83%	47.17%	50		
	Never or almost never	18.75%	81.25%	32		
Milk and natural dairy drinks	Several times a day	32.25%	68.75%	16	0.0001 **	45.33
(e.g., milk, milk soups, natural yogurt, kefir, natural buttermilk, etc.)	Daily	36.05%	63.95%	86		
	Several times a week	60.00%	40.00%	95		
	Several times a month	75.86%	24.14%	87		
	Once a month or less	64.00%	36.00%	25		
	Never or almost never	78.57%	21.43%	14		
Eggs and dishes where eggs are	Several times a day	100.00%	0.00%	2	0.7667	21.47
the main ingredient (e.g.,	Daily	42.86%	57.14%	42		
scrambled	Several times a week	61.54%	38.46%	130		
eggs, omelet, egg pasta,	Several times a month	61.26%	38.74%	111		
boiled eggs, etc.)	Once a month or less	41.94%	58.06%	31		
	Never or almost never	66.67%	33.33%	6		
Whole grain or seeded bread,	Several times a day	60.00%	40.00%	5	0.0168 **	26.32
also known as dark bread (e.g.,	Daily	50.94%	49.06%	53		
rye whole grain bread, graham bread, wheat or rye bread with seeds, pumpernickel, etc.)	Several times a week	69.39%	30.61%	98		
	Several times a month	64.13%	35.87%	92		
	Once a month or less	40.00%	60.00%	55		
	Never or almost never	35.00%	65.00%	20		
Refined bread, commonly known as white bread (e.g.,	Several times a day	71.43%	28.57%	7	0.00001 **	28.64
white wheat	Daily	63.27%	36.73%	49		
or rye bread, wheat-rye bread,	Several times a week	74.00%	26.00%	100		
toast bread, plain rolls, buns and croissants, etc.)	Several times a month	61.64%	38.36%	73		
	Once a month or less	34.38%	65.63%	64		
	Never or almost never	30.00%	70.00%	30		
Oils, all types	Several times a day	12.50%	87.50%	8	0.00001 **	33.67
	Daily	14.49%	85.51%	69		
	Several times a week	61.00%	39.00%	100		
	Several times a month	73.12%	26.88%	93		
	Once a month or less	85.37%	14.63%	41		
	Never or almost never	91.67%	8.33%	12		
Butter, all types	Several times a day	100.00%	0.00%	7	0.00001 **	84.21
	Daily	87.10%	12.90%	31		
	Several times a week	79.41%	20.59%	68		
	Several times a month	54.95%	45.05%	91		
	Once a month or less	29.23%	70.77%	65		
	Never or almost never	47.54%	52.46%	61		
Fruits, all types	Several times a day	48.48%	51.52%	33	0.0061 **	41.33
	Daily	50.00%	50.00%	84		
	Several times a week	71.88%	28.13%	128		
	Several times a month	48.39%	51.61%	62		
	Once a month or less	50.00%	50.00%	12		
	Never or almost never	0.00%	100.00%	4		
Vegetables, all types	Several times a day	43.48%	56.52%	23	0.04501 **	44.23
	Daily	59.60%	40.40%	99		
	Several times a week	64.29%	35.71%	126		
	Several times a month	53.57%	46.43%	56		
	Once a month or less	41.67%	58.33%	12		
	Never or almost never	14.29%	85.71%	7		
Red meat (e.g., pork, beef, veal, etc.)	Daily	46.67%	53.33%	15	0.00158 **	33.21
	Several times a week	53.42%	46.58%	73		
	Several times a month	51.08%	48.92%	139		
	Once a month or less	62.07%	37.93%	58		
	Never or almost never	86.84%	13.16%	38		
Poultry and rabbit meat (e.g., poultry meat from chicken, hen, duck, turkey, etc.)	Several times a day	0.00%	100.00%	1	0.01187 **	11.23
	Daily	48.00%	52.00%	25		
	Several times a week	57.55%	42.45%	139		
	Several times a month	52.21%	47.79%	113		
	Once a month or less	63.64%	36.36%	22		
	Never or almost never	91.30%	8.70%	23		
Fatty fish (e.g., salmon, sardines, herring, mackerel, large carp, eel, etc.)	Several times a week	57.14%	42.86%	14	0.1455	22.84
	Several times a month	67.53%	32.47%	77		
	Once a month or less	57.72%	42.28%	123		
	Never or almost never	50.46%	49.54%	109		

* Pearson chi-squared test; ** Significant dependencies at the α = 0.05 level.

**Table 4:** Characteristics of the frequency of consumption of selected beverages among participants.

Food Products		Country		Total N	*p* *	chi^2^
		Poland	Greece			
Vegetable and vegetable–fruit	Never or almost never	51.75%	48.25%	114	0.01101 **	47.63
juices (e.g., mixed vegetable,	Once a month or less	66.67%	33.33%	90		
tomato, carrot, carrot-fruit, etc.)	Several times a month	62.67%	37.33%	75		
	Several times a week	55.88%	44.12%	34		
	Daily	12.50%	87.50%	8		
	Several times a day	0.00%	100.00%	2		
Sweetened beverages	Never or almost never	44.87%	55.13%	78	0.00052 **	55.01
(e.g., Fanta, Coca-Cola, Mirinda, Sprite, etc.)	Once a month or less	48.15%	51.85%	108		
	Several times a month	70.51%	29.49%	78		
	Several times a week	74.42%	25.58%	43		
	Daily	78.57%	21.43%	14		
	Several times a day	50.00%	50.00%	2		
Beer	Never or almost never	52.08%	47.92%	96	0.00178 **	22.97
	Once a month or less	50.00%	50.00%	96		
	Several times a month	74.49%	25.51%	98		
	Several times a week	48.00%	52.00%	25		
	Daily	37.50%	62.50%	8		
Wine and cocktails	Never or almost never	74.58%	25.42%	59	0.007123 **	33.36
	Once a month or less	53.28%	46.72%	137		
	Several times a month	60.00%	40.00%	100		
	Several times a week	34.78%	65.22%	23		
	Daily	0.00%	100.00%	2		
	Several times a day	50.00%	50.00%	2		
Vodka and high-proof spirits	Never or almost never	52.75%	47.25%	91	0.74392	44.97
	Once a month or less	58.39%	41.61%	137		
	Several times a month	62.20%	37.80%	82		
	Several times a week	50.00%	50.00%	10		
	Daily	66.67%	33.33%	3		

* Pearson chi-squared test; ** Significant dependencies at the α = 0.05 level.

**Table 5:** Distribution of study participants in the different ranges of the healthy diet index pHDI-10.

Pro-Healthy Diet Index (pHDI-10)	Number	Percentage
Low	309	95.67
Moderate	14	4.33
High	0	0

**Table 6:** Distribution of the study participants in the different ranges of healthy diet index pHDI-10 with the division between Polish and Greek students.

Pro-Healthy Diet Index (pHDI-10)	Country		Total N	df	*p* *	chi^2^
	Poland	Greece				
Low	57.93%	42.07%	309	1	0.5571	142.31
Modetate	50.00%	50.00%	14			
High	0.00%	0.00%	0			
Total	186	137	323			

* Pearson chi-squared test.

**Table 7:** Distribution of study participants in the different ranges of the healthy diet index pHDI-10 in terms of nutritional status.

Poland											
**BMI**	Pro-Healthy Diet Index (pHDI-10)			Total N			df	*p* *	chi^2^		
	low	moderate	high								
**Normal BMI [%]**	96.62%	3.38%	0.00%	148		2		0.65656	54.23		
**Overweight/obese [%]**	93.75%	6.25%	0.00%	32							
**underweight [%]**	100.00%	0.00%	0.00%	6							
**Total**	179	7	0	186							
* Pearson chi-squared test											
**Greece**											
**BMI**		Pro-Healthy Diet Index (pHDI-10)					Total N	df	*p* *	chi^2^	
	low		moderate		high						
**Normal BMI [%]**	94.74%		5.26%		0.00%		114	2	0.80871	65.33	
**Overweight/obese [%]**	93.75%		6.25%		0.00%		16				
**Underweight [%]**	100.00%		0.00%		0.00%		7				
**Total**	130		7		0		137				
* Pearson chi-squared test											

## Data Availability

All data are stored with the authors of the publication and can be made available upon request.
